# Self-reported Rates of Abuse, Neglect, and Bullying Experienced by Transgender and Gender-Nonbinary Adolescents in China

**DOI:** 10.1001/jamanetworkopen.2019.11058

**Published:** 2019-09-06

**Authors:** Ke Peng, Xuequan Zhu, Amy Gillespie, Yuanyuan Wang, Yue Gao, Ying Xin, Ji Qi, JianJun Ou, Shaoling Zhong, Lixian Zhao, Jianbo Liu, Chaoyue Wang, Runsen Chen

**Affiliations:** 1 National Clinical Research Center for Mental Disorders, Beijing Key Laboratory of Mental Disorders and Advanced Innovation Center for Human Brain Protection, Beijing Anding Hospital, Capital Medical University, Beijing, China; 2 Department of Psychiatry, University of Oxford, Oxford, United Kingdom; 3 Division of Psychology, Faculty of Health and Life Sciences, De Montfort University, Leicester, United Kingdom; 4 Beijing LGBT Center, Beijing, China; 5 Department of Psychiatry and Mental Health Institute, Second Xiangya Hospital, Central South University, Chinese National Clinical Research Centre on Mental Disorders, Changsha, China; 6 First Affiliated Hospital of Guangzhou University of Traditional Chinese Medicine, Guangzhou, China; 7 Department of Child Psychiatry, Shenzhen Kangning Hospital, Shenzhen Mental Health Center, Shenzhen, China; 8 Nuffield Department of Clinical Neurosciences, University of Oxford, Oxford, United Kingdom

## Abstract

**Importance:**

This is the first comprehensive national study reporting the rates of abuse, neglect, and bullying from family and classmates or teachers among Chinese transgender and gender-nonbinary adolescents and identifying risk factors associated with poor mental health in this population.

**Objective:**

To assess the rates of abuse, neglect, and bullying and their association with poor mental health among Chinese transgender and gender nonbinary adolescents.

**Design, Setting, and Participants:**

This national survey study used an online self-selecting survey conducted between January 1, 2017, and September 29, 2017, in China. Eligibility criteria included reporting being aged 12 to 18 years and being transgender or gender nonbinary. Data analysis was performed from March 25 to 28, 2019.

**Main Outcomes and Measures:**

The main outcome was self-reported poor mental health, including depressive symptoms, anxiety symptoms, and suicidal ideation. Depressive symptoms were measured using the Center for Epidemiological Studies Depression 9-item scale. Anxiety symptoms were measured using the 7-item General Anxiety Disorder scale. Suicidal ideation was measured using standardized questions adapted from previous Chinese studies. Abuse, neglect, and bullying were measured using specifically designed questions.

**Results:**

Of 564 responses collected, 385 respondents (mean [SD] age, 16.7 [1.2] years) met inclusion criteria, including 109 (28.3%) transgender adolescent boys, 167 (43.4%) transgender adolescent girls, and 109 (28.3%) gender-nonbinary adolescents. Among 319 respondents who reported that their parents were aware of their gender identity, 296 (92.8%) reported having experienced parental abuse or neglect. Among the full cohort, 295 respondents (76.6%) reported having experienced abuse or bullying owing to being transgender or gender nonbinary in school from classmates or teachers. There were 173 respondents (44.9%) with Center for Epidemiological Studies Depression 9-item scale scores indicating they were at risk of major depressive disorder, and 148 respondents (38.4%) had 7-item General Anxiety Disorder scale scores indicating they were at risk of an anxiety disorder. In univariate analysis, reporting experiences of bullying from a classmate or teacher was significantly associated with suicidal ideation (odds ratio, 1.68 [95% CI, 1.04-2.70]; *P* = .03), but the association was no longer statistically significant after controlling for level of educational attainment, aversion to assigned sex, and depressed mood at the onset of puberty (odds ratio, 1.63 [95% CI, 0.97-2.73]; *P* = .06).

**Conclusions and Relevance:**

In this survey study, transgender and gender-nonbinary adolescents in China reported high rates of abuse, neglect, and bullying at home and in school and high rates of symptoms associated with poor mental health. This study highlights the importance of reducing home- and school-based abuse, neglect, and bullying of transgender and gender-nonbinary adolescents in China to improve mental health outcomes; however, broader change in the social environment may be required to address the prejudice and stigma aimed at gender minorities.

## Key Points

**Question:**

What are the rates of abuse, neglect, and bullying and their association with poor mental health experienced by Chinese transgender and gender nonbinary adolescents?

**Findings:**

In this national survey study of 385 transgender and gender-nonbinary adolescents, 296 respondents reported experiencing parental abuse or neglect and 295 respondents reported experiencing abuse or bullying from classmates or teachers. Reporting experiences of bullying or abuse from classmates or teachers was significantly associated with increased risk of suicidal ideation.

**Meaning:**

Greater access to appropriate mental health care may be helpful to address the high rates of mental health issues and abuse, neglect, and bullying experienced by Chinese transgender and gender-nonbinary adolescents.

## Teaser

This survey study assesses the rates of abuse, neglect, and bullying experienced at home or in school by transgender and gender-nonbinary adolescents in China and the associations of these experiences with mental health.

## Introduction

Social awareness of adolescent experiences of abuse, neglect, and bullying at home and in school has increased in the last few decades, with acknowledgment that child abuse has been a long-lasting and severe phenomenon in China.^1,2^ One national survey of 3543 adults in China found that 71.9% of Chinese adults reported being abused by their parents during childhood.^3^ However, a multiprovince survey in China reported 1-year incidence rates of 26.1% for bullying, 9.0% for bullying others, and 28.9% for witnessing bullying,^4^ which are equivalent to rates reported in North America; one US study^5^ found that 28% of youths reported being bullied by others in the past 6 months and 20% of youths reported being bullied multiple times.

The mental health implications for Chinese adolescents of these high rates of abuse, neglect, and bullying at home and in school warrant further attention, particularly the implications for suicidal behavior. Suicide was reported to be the fourth leading cause of death for adolescents in China in 2016,^6,7^ with a 2010 national survey^8^ reporting that 2.7% of Chinese adolescents had made a suicide attempt in the past 12 months and 0.6% of adolescents had made multiple attempts. A 2002 study^9^ reported that more than half of Chinese adolescents who reported suicidal ideation had attempted suicide in the past 12 months. Abuse, neglect, and bullying at home and in school have been found to increase the risk of suicidal ideation and attempts among adolescents.^10^

*Sexual and gender minority* is an umbrella phrase that encompasses lesbian, gay, bisexual, transgender, and intersex populations, as well as those whose sexual orientation, gender identity and expressions, or reproductive development vary from traditional, societal, cultural, or physiological norms. Specifically, the term *sexual minorities* includes individuals whose sexual identity, orientation, or practices vary from heterosexual norms.^11,12^ The term *gender minorities* includes individuals who do not identify with some, or all, of the aspects of the sex they were assigned at birth, such as identifying as transgender, nonbinary, or genderqueer.^13^

In the United States, it has been reported that gender-based abuse, neglect, and bullying are more common for adolescents who are sexual or gender minorities than for heterosexual cisgender adolescents,^14^ with most sexual or gender minorities reporting experiences of abuse, neglect, and bullying.^15^ A lack of acceptance from parents and unfriendly school environments for sexual or gender minority students, which are reported to be more severe in China, are significant contributors to experiences of abuse, neglect, and bullying.^16,17,18,19,20,21,22^ Furthermore, a 2013 study from the United States reported that rates of suicidal ideation and suicide attempts among transgender adolescents were 2-fold those of their cisgender peers.^23^ Barriers for obtaining hormone interventions and associated mental health concerns may increase the risk of suicidal ideation and suicide attempts.^24^

Minority stress theory is a conceptual framework that has been applied to numerous population groups to understand the association of a hostile and stressful social environment involving stigma, prejudice, and discrimination with poor mental health outcomes.^25,26^ Prejudice creates a noxious environment for minority groups,^27^ and discrimination and violence driven by prejudice have been shown to increase stressors for minority groups with a subsequent negative effect on mental well-being^26^; in contrast, social support has been shown to be an independent factor associated with better mental health.^22,28^ In the last decade, the minority stress theory has been used to explore mental health concerns in transgender populations and assist clinicians in treating transgender patients in the context of societal hostility.^26,29^

However, to our knowledge, the rates of abuse, neglect, and bullying among specifically Chinese transgender or gender-nonbinary adolescents are not well documented, nor are the rates of suicidal ideation or behavior. It is important to understand the rates of abuse, neglect, bullying, and suicidal ideation among Chinese transgender adolescents and to develop specific preventative interventions to target this form of minority stress. Therefore, this study aimed to explore the rates of parental abuse and neglect and classmate or teacher abuse or bullying experienced by transgender and gender-nonbinary adolescents owing to their gender minority status in the Chinese population and to investigate whether these experiences were associated with suicidal ideation or other self-reported poor mental health (ie, depressive or anxiety disorders).

## Methods

Full details of the study procedures and sample have been previously published^30,31^; the full sample included transgender and gender-nonbinary adults. Our previous 2 publications included a report on barriers that Chinese transgender adults faced when accessing health care and gender-affirming interventions^30^ and a report on suicidal ideation and attempt rates among Chinese transgender adults.^31^ These studies identified numerous barriers to accessing health care and gender-affirming treatment, high rates of suicidal ideation, and associations of suicidal ideation and suicide attempts with education level, relationship status, parental conflict, self-harm, and seeking mental health services.^31^ This study focused on adolescents and specific experiences of abuse, neglect, and bullying at home or in school.

Briefly, a survey study using self-selected samples was conducted between January 1, 2017, and September 29, 2017. Convenience sampling, respondent-driven sampling, and snowball sampling methods that have previously proven to be effective methods for recruiting lesbian, gay, bisexual, or transgender participants were used to recruit participants. Questionnaires were distributed around the country via lesbian, gay, bisexual, or transgender service centers, educational institutes, social media, telephone calls, text messages, and online recruitment advertisements (phase 1). Initial participants were invited to distribute the questionnaire to their transgender and gender-nonbinary friends and acquaintances (phase 2). All questionnaires were anonymous. Participants completed an online survey questionnaire that collected information about demographic characteristics, including gender identity, housing status, employment status, relationship status, children, and suicidal behaviors. The inclusion criteria were having a distinct IP address, completing more than 85% of the questionnaire, spending a minimum of 8 minutes completing the survey (in a pilot study, 8 minutes was the minimum time recorded for completing 85% of the questionnaire; the mean survey completion time was 35 minutes), and meeting the definitions used in the study for either transgender adolescent boys, transgender adolescent girls, or gender-nonbinary adolescents.^31^ To assess gender identity, we asked participants their assigned biological sex at birth and their self-identified gender. Participants who self-identified as female and were assigned male at birth were categorized as transgender adolescent girls, and participants who self-identified as male and were assigned as female at birth were categorized as transgender adolescent boys. Participant who were uncertain about self-identified gender were categorized as gender-nonbinary adolescents.

A version of the Center for Epidemiological Studies Depression 9-item (CES-D-9) scale modified for use with a Chinese population^32^ was used to screen for depressive symptoms, with a total score of 17 points or higher indicating risk for major depressive disorder. The CES-D-9 scale has been validated in a Chinese population and shown good reliability (Cronbach α = 0.85-0.88). The Chinese-validated version of the 7-item General Anxiety Disorder scale was used to screen for anxiety symptoms, with a total score of 10 points or higher indicating risk for anxiety disorder. The 7-item General Anxiety Disorder scale has also shown good reliability in a Chinese population (Cronbach α range, 0.76-0.83).^33^ Suicidal ideation was measured using standardized questions with yes or no options, which have been used in previous Chinese research.^33^ Abuse, neglect, and bullying owing to being transgender or gender nonbinary that were experienced at home from family members or in school from classmates or teachers were assessed using questions such as, “Have your parents or guardians limited your personal freedom? [in Chinese]” for the home aspect and “Have your classmates or teachers mocked you in public? [in Chinese]” for the school aspect (eTable in the Supplement). For parental abuse and neglect experiences, a question asked respondents if their family was aware of their transgender or nonbinary identity. If respondents indicated that their family was unaware of their gender identity, they were not asked about their parents or guardians exposing them to abuse or neglect owing to being transgender or nonbinary, as it would not have been possible for them to attribute with certainty the abuse or neglect to their gender identity. All results regarding abuse or neglect from parents or guardians refer to the remaining participants whose families were aware of their gender identities.

Ethical approval was obtained from Beijing Anding Hospital, Capital Medical University, and all participants provided either written informed consent or assent online. Owing to the uniqueness of the study sample, informed consent could not be obtained from the parents of the participants who were younger than 18 years. Additionally, some parents of the participants were unaware of the gender identity of their children; therefore, it was not possible to obtain their consent. The ethical review approved of this change to informed consent requirements.

For this study, we only included participants aged 12 to 18 years who identified as transgender or gender nonbinary. As this sample was self-selected and the number of people exposed to the survey is unknown, we cannot report response rates; this is in line with American Association for Public Opinion Research (AAPOR) reporting guidelines for self-selected samples, which were used to report this study.

### Statistical Analysis

Data analysis was performed from March 25 to 28, 2019. We reported the characteristics of the participants and reported rates of abuse, neglect, and bullying experiences and poor mental health using descriptive statistics, categorical variables with percentages, and continuous variables with means and SDs as appropriate. Logistic regression was performed to analyze univariate associations of social or demographic factors with lifetime suicidal ideation among transgender and gender-nonbinary participants. Variables that were significantly associated with suicidal ideation in the univariate analysis were entered into a multivariate logistic regression model to identify factors associated with suicidal ideation, and odds ratios (ORs) and 95% CIs were used to report the results of logistic analysis. Data were analyzed using SAS statistical software version 9.4 (SAS Institute). *P* values were 2-sided and statistical significance was set at less than .05.

## Results

A total of 564 responses were received, and 385 (68.2%) respondents were included after screening against the core inclusion and exclusion criteria. Sixty-six respondents indicated that their families were unaware of their gender identities; therefore, these individuals did not answer questions regarding abuse or neglect from parents or guardians. Participants were aged 12 to 18 years (mean [SD] age, 16.7 [1.2] years), and 323 participants (83.9%) were students. Respondents included 109 (28.3%) transgender adolescent boys, 167 (43.4%) transgender adolescent girls, and 109 (28.3%) gender-nonbinary adolescents.

Among the full cohort, 319 respondents (82.9%) reported that their family was aware of their gender identity and therefore answered questions on parental abuse and neglect. Among these respondents, 296 (92.8%) reported exposure to parental abuse or neglect owing to being transgender or gender nonbinary at some point in their lifetime, and 282 respondents (88.4%) reported experiences of abuse, neglect, or bullying in the past 12 months. No statistically significant differences were found in the rates of lifetime suicidal ideation between respondents who reported experiencing parental abuse or neglect compared with those who did not (OR, 1.57 [95% CI, 0.67-3.70]). A further 217 respondents (68.0%) reported experiencing parental abuse or neglect more than 3 times in the past 12 months. Lifetime experiences of economic control were reported by 211 respondents (66.1%), of persistent neglect by 140 respondents (43.9%), of verbal abuse or insults by 191 respondents (59.9%), and of restriction of personal freedom by 162 respondents (50.8%). At least 1 experience of physical abuse was reported by 151 respondents (47.3%), and 60 respondents (18.8%) reported being forced out of the home or family members cutting off contact (Table 1).

**Table 1.** Respondents Who Experienced Each Type of Abuse, Bullying, or Neglect From Parent or Classmate or Teacher

Type	No. (%)
Parental abuse or neglect, No./total No. (%)	296/319 (92.8)
Economic control	211 (66.1)
Being forced to change gender expression	202 (63.3)
Deliberately neglecting or evading gender identity and giving no care or support	202 (63.3)
Verbal abuse or insults	191 (59.9)
Restricting personal freedom	162 (50.8)
Physical assault	151 (47.3)
Persistent neglect	140 (43.9)
Eviction from the home or cutting off contact	60 (18.8)
Being coerced or forced to undergo conversion therapy	55 (17.2)
Being forced to have sex with others	3 (0.9)
Classmate or teacher abuse and bullying, No./total No. (%)	295/385 (76.6)
Verbal bullying	264 (68.6)
Being publicly mocked	80 (20.7)
Isolation or exclusion	190 (49.4)
Spread of rumors	192 (49.9)
Threats or intimidation	113 (29.4)
Cyberbullying via social media	248 (64.4)
Physical abuse	149 (38.7)

From the full cohort, 295 respondents (76.6%) reported bullying or abuse owing to being transgender or gender nonbinary from classmates or teachers. We found statistically significant differences in rates of lifetime suicidal ideation between youths who had experienced classmate or teacher abuse or bullying compared with those who had not (OR, 1.68 [95% CI, 1.04-2.70]). The most commonly reported form of abuse or bullying in school was verbal bullying (264 respondents [68.6%]). Being publicly mocked (80 respondents [20.7%]) and threats or intimidation (113 respondents [29.4%]) were the least commonly reported, but they were still reported by more than 20% of respondents (Table 1).

Among the full cohort, 173 respondents (44.9%) were found to be at risk of major depressive disorder, and 148 respondents (38.4%) were found to be at risk of an anxiety disorder. Higher rates of mental health problems and suicidal behaviors were reported by transgender adolescent girls (mean [SD] CES-D-9 score, 15.7 [6.0] points) compared with transgender adolescent boys (mean [SD] CES-D-9 score, 14.2 [7.8] points) and gender nonbinary adolescents (mean [SD] CES-D-9 score, 14.3 [6.7] points). Compared with gender nonbinary adolescents, transgender adolescent girls were more likely to be at risk of major depressive disorder (OR, 1.4 [95% CI, 0.9-2.2]), but the difference for transgender adolescent boys was nonsignificant (OR, 1.0 [95% CI, 0.6-1.7]). Transgender adolescent boys and girls had higher rates of disliking their assigned sex (transgender boys: OR, 2.5 [95% CI, 1.4-4.5]; transgender girls: OR, 5.5 [95% CI, 3.0-9.9]), feeling pain and depression at the onset of puberty (transgender boys: OR, 3.5 [95% CI, 1.8-6.9]; transgender girls: OR, 6.1 [95% CI, 3.1-12.1]), wanting to stop puberty (transgender boys: OR, 3.9 [95% CI, 1.8-8.2]; transgender girls: OR, 4.1 [95% CI, 2.1-7.9]), and wanting access to hormone intervention (transgender boys: OR, 4.7 [95% CI, 2.5-8.7]; transgender girls: OR, 10.7 [95% CI, 5.6-20.5]) compared with gender nonbinary adolescents. Across the sample, 196 respondents (50.9%) reported suicidal ideation, with the highest rates among transgender adolescent girls (107 of 167 respondents [64.1%]) followed by transgender adolescent boys (53 of 109 respondents [48.6%]). A total of 61 respondents (15.8%) reported a suicide attempt at some point in their lifetime.

The results of the univariate analyses are shown in Table 2. Compared with participants who attained a college degree or higher, those with only junior school education or below were more likely to report suicidal ideation (OR, 2.43 [95% CI, 1.24-4.75]; *P* = .009). Suicidal ideation was also more likely to be reported by participants who strongly disliked their assigned sex (OR, 4.98 [95% CI, 2.93-8.47]; *P* < .001) and participants who felt pain and depressed mood at the onset of puberty (OR, 7.44 [95% CI, 3.66-15.13]; *P* < .001). In univariate analysis, having experienced abuse or bullying from classmates or teachers was also found to be significantly associated with suicidal ideation (OR, 1.68 [95% CI, 1.04-2.70]; *P* = .03). However, in the multivariate model using stepwise regression, abuse or bullying from classmates or teachers was no longer associated with suicidal ideation after the model was adjusted for level of educational attainment, strong dislike of assigned sex, and feelings of pain and depressed mood at the onset of puberty (OR, 1.63 [95% CI, 0.97-2.73]; *P* = .06) (Table 3).

**Table 2.** Univariate Analysis of Factors Associated With Suicidal Ideation

Variable	Lifetime Suicidal Ideation, No. (%) (n = 196)	Odds Ratio (95% CI)	*P* Value
Age, y			.25
≤15	29 (43.9)	1 [Reference]	NA
16	40 (59.7)	1.89 (0.95-3.76)	.09
17	67 (53.2)	1.45 (0.80-2.64)	.62
18	60 (47.6)	1.16 (0.64-2.11)	.40
Education status			.97
Currently at school	164 (50.7)	1 [Reference]	NA
Graduated	14 (50.0)	0.97 (0.45-2.10)	.86
Dropped out of school	18 (52.9)	1.09 (0.54-2.21)	.80
Education			.03
Bachelor’s degree and higher	19 (37.3)	1 [Reference]	NA
High school	105 (49.5)	1.65 (0.88-3.10)	.79
Junior school and lower	72 (59.0)	2.43 (1.24-4.75)	.009
Highest level of education of parents			.66
Junior school and lower	43 (51.8)	1 [Reference]	NA
High school	50 (47.2)	0.83 (0.47-1.48)	.39
Bachelor’s degree and higher	103 (52.6)	1.03 (0.62-1.72)	.55
Sexual orientation			.44
Heterosexual	37 (57.8)	1 [Reference]	NA
Homosexual	33 (45.8)	0.62 (0.31-1.22)	.38
Bisexual or pansexual	101 (52.1)	0.79 (0.45-1.40)	.65
Asexual, uncertain, or other	25 (45.5)	0.61 (0.29-1.26)	.38
Strongly dislike assigned sex	174 (60.0)	4.98 (2.93-8.47)	<.001
Felt pain and depressed mood at the onset of puberty	186 (57.9)	7.44 (3.66-15.13)	<.001
Ever experienced bullying or abuse in school	159 (53.9)	1.68 (1.04-2.70)	.03
Ever experienced abuse, bullying, or neglect from family^a^	162 (54.7)	1.57 (0.67-3.70)	.30
Experienced abuse, bullying, or neglect from family in past 12 mo^b^	131 (60.4)	2.36 (1.39-4.00)	.001

Abbreviation: NA, not applicable.

^a^ Includes 319 respondents.

^b^ Includes 296 respondents.

**Table 3.** Analysis of Factors Associated With Suicidal Ideation in Stepwise Logistic Regression

Adjustment	Odds Ratio (95% CI)	*P* Value
Model 1^a^	1.68 (1.04-2.70)	.03
Model 2^b^	1.62 (1.01-2.63)	.04
Model 3^c^	1.63 (0.97-2.73)	.06

^a^ Univariate analysis including abuse and bullying in school only.

^b^ Model 1 with adjustment for educational attainment.

^c^ Model 2 with adjustment for strong dislike of assigned sex and pain and depressed mood at the onset of puberty.

## Discussion

This study found that Chinese transgender or gender-nonbinary adolescents reported a high rate (92.8%) of parental abuse and neglect, with nearly all respondents reporting having experienced some form of abuse, neglect, or bullying owing to being transgender or gender nonbinary. The high rates of parental abuse and neglect may in part be due to the socially conservative values prevalent in China, meaning that not only do parents struggle to accept their children being transgender or gender nonbinary, resulting in insufficient family support, but parents attempt to discourage or punish their children through control and psychological abuse. This treatment may contribute to distal minority stress processes, which may exacerbate proximal stressors,^31^ whereas positive parental support for transgender children has been found to be significantly associated with better mental health outcomes.^34^

Similarly, 76.6% of participants reported experiencing abuse or bullying from classmates or teachers, and this experience was associated with suicidal ideation among transgender or gender-nonbinary adolescents. In a similar North American study of transgender adolescents,^14^ a lower rate of abuse and bullying by classmates or teachers was reported (44.8%), but the study also identified a significant association of classmate or teacher abuse and bullying with suicide attempts. A study by Pedro and Esqueda^35^ reported that the rate of physical or nonphysical abuse or bullying among transgender adolescents was nearly double the rate among their cisgender counterparts. These common experiences of abuse, neglect, and bullying may be the main contributor to high rates of poor mental health in transgender adolescents.^36,37^ Compared with cisgender adolescents, transgender adolescents have reported receiving less support from the school and more vulnerability to mistreatment, even receiving harassment from school staff members.^36^ A lower sense of belonging among the school community has been reported among sexual minority students^38^ and among those who experience bullying from peers,^39^ and there may be similar findings for transgender or gender-nonbinary students owing to societal prejudice and stigma. Being accepted and respected by peers in school is associated with a decreased risk of abuse and bullying by classmates or teachers,^40,41^ and feeling a sense of belonging may contribute to improved educational achievement and reduced risk of poor mental health outcomes.^42,43^

In line with the gender minority stress model that gives a framework for an association of a hostile and stressful social environment for minority groups with the mental health of individuals within a minority group,^25,26^ we also found high reported rates of poor mental health, consistent with findings from Western countries.^26,44,45,46,47^ The rates of self-reported mental health symptoms that are indicative of risk of major depressive disorder and anxiety disorders were 44.9% and 38.4%, respectively. In a 1999 general population study of Chinese adolescents,^48^ the rate of self-reported depression was 16.9%, indicating that transgender or gender-nonbinary adolescents report depression at rates 2- to 3-fold those of the general adolescent population. This is consistent with findings from a previous retrospective cohort study conducted in Boston, Massachussetts,^49^ that included a sample of 180 transgender and 180 cisgender adolescents. That study by Reisner et al^49^ reported the rate of depression among the transgender adolescents as 50.6% compared with 20.6% in the cisgender group, indicating a similar ratio of rates 2- to 3-fold higher for transgender adolescents. Considering the greater burden of symptoms of poor mental health, our finding of high rates of suicidal ideation was unsurprising. We found the rate of lifetime suicidal ideation among transgender and gender-nonbinary adolescents to be 50.9%, and among participants who reported suicidal ideation, 31.1% of them had made at least 1 suicide attempt. A 2013 study^23^ reported that the prevalences of suicidal ideation and suicide attempts in cisgender adolescents in the United States were 12.1% and 4.1%, respectively, with the rate of suicidal ideation found to be nearly 2-fold higher among transgender adolescents compared with their cisgender counterparts.^50^

In summary, our results indicate that sociocultural prejudice against transgender and gender nonbinary individuals in China was expressed through high rates of abuse, neglect, and bullying against adolescents, both in school and at home, and that these adolescents experienced high rates of suicidal ideation and high risk of poor mental health. The environment for gender minorities in China appears to be hostile. There is a lack of social support in the 2 primary sources of social interaction, home and school, and transgender and gender-nonbinary individuals frequently experience poor mental health. Expected statistical associations of experiences of abuse, neglect, and bullying with mental health outcomes were minimal, although this may be because experiences of abuse, neglect, and bullying were common to nearly every respondent in the sample, creating a ceiling effect in the data. However, other studies have reported these associations, such as a 2008 study^28^ in sexual minorities in the United States and a 2018 study^22^ in Chinese sexual minorities.

These findings of increased abuse, neglect, bullying, and their association with poor mental health in transgender and gender-nonbinary Chinese adolescents suggest an urgent need for numerous changes on a several levels. Development of comprehensive mental health interventions in China to improve the psychological well-being of transgender or gender-nonbinary adolescents and reduce the risk of suicide should be researched and implemented, with consideration for the effects of abuse, neglect, and bullying experienced at home and in school. Furthermore, schools should develop evidence-based plans for preventing abuse, neglect, and bullying of transgender and gender-nonbinary students, such as staff training sessions and educational programs for students, focused on understanding and respecting gender and sexual diversity. This could contribute to creating a less hostile school environment for transgender and gender-nonbinary adolescents, reducing sources of stress. The Chinese government could also consider implementing education for parents regarding support for transgender and gender-nonbinary children and interventions to reduce parental abuse and neglect. Research to establish the most effective interventions for schools and families would be of great benefit.

### Limitations

While this is the first national-level survey exploring abuse, neglect, and bullying experienced by transgender and gender-nonbinary youth and the association of abuse, neglect, and bullying with suicide ideation in China to our knowledge, there are limitations to this study. As the study sample was recruited from the national level, questionnaires were only accessible to adolescents with internet access; therefore, the sample may not be representative, and it may be not appropriate to generalize the findings to economically underdeveloped areas without internet access. The sampling methods (ie, convenience sampling, respondent-driven sampling, and snowball sampling), while effective at obtaining a large sample, may also bias findings and do not allow us to infer population rates. There are numerous variables (eg, physical exercise) that may confound the association of abuse and bullying by classmates or teachers with suicidal ideation but that we did not collect. All our measures were self-reported and conducted over the internet, so we were unable to have clinicians assess whether respondents met clinical criteria for diagnoses. For parental abuse and neglect, the issue of missing data could affect the accuracy of results. Additionally, experiences of abuse, neglect, and bullying were not measured using a recognized or standardized scale, so, although we used expert consultation to refine the questions, the reliability and validity of the abuse, neglect, and bullying measure has not been examined.

## Conclusions

Transgender and gender-nonbinary adolescents in China face significant challenges in their social environments. In the first national online survey conducted to assess this, to our knowledge, we found very high self-reported rates of abuse, neglect, and bullying from family members and in school among Chinese transgender and gender-nonbinary adolescents. Self-reported symptoms indicative of poor mental health were also very common among transgender and gender nonbinary adolescents, as was suicidal ideation. Our findings suggest that abuse and bullying by classmates or teachers specifically may contribute to suicidal ideation. Future research should confirm and expand on these findings; in particular, a more representative sample should be studied using validated questionnaires.

## Notes

Supplement. **eTable.** Survey Questions on Abuse, Neglect, and Bullying at Home and in School, Translated From Chinese

## References

[1] Qiao D, Chan Y-C Child abuse in China: a yet-to-be-acknowledged ‘social problem’ in the Chinese mainland. Child Fam Soc Work. 2005;10(1):-. doi:10.1111/j.1365-2206.2005.00347.x

[2] Kwok SY, Chai W, He X Child abuse and suicidal ideation among adolescents in China. Child Abuse Negl. 2013;37(11):986-996. doi:10.1016/j.chiabu.2013.06.006 23899534

[3] Liu M, Zhang L Actuality, attitude and intervention: a survey report about family violence against women In: Rong W, ed. Combating Family Violence Against Women: Chinese Theories and Practices. Beijing, China: China Social Sciences Press; 2002.

[4] Han Z, Zhang G, Zhang H School bullying in urban China: prevalence and correlation with school climate. Int J Environ Res Public Health. 2017;14(10):e1116. doi:10.3390/ijerph14101116 28946682 PMC5664617

[5] Dinkes R, Cataldi EF, Lin-Kelly W Indicators of School Crime and Safety: 2007. NCES 2008-021/NCJ 219553. Jessup, MD: National Center for Education Statistics; 2007.

[6] Xu RB, Wen B, Song Y, The change in mortality and major causes of death among Chinese adolescents from 1990 to 2016 [in Chinese]. Zhonghua Yu Fang Yi Xue Za Zhi. 2018;52(8):802-808. doi:10.3760/cma.j.issn.0253-9624.2018.08.006 30107713

[7] Chen R, An J, Ou J Suicidal behaviour among children and adolescents in China. Lancet Child Adolesc Health. 2018;2(8):551-553. doi:10.1016/S2352-4642(18)30170-6 30119712

[8] Xing X-Y, Tao F-B, Wan Y-H, Family factors associated with suicide attempts among Chinese adolescent students: a national cross-sectional survey. J Adolesc Health. 2010;46(6):592-599. doi:10.1016/j.jadohealth.2009.12.006 20472217

[9] Hesketh T, Ding QJ, Jenkins R Suicide ideation in Chinese adolescents. Soc Psychiatry Psychiatr Epidemiol. 2002;37(5):230-235. doi:10.1007/s00127-002-0536-9 12107715

[10] Rigby K, Slee P Suicidal ideation among adolescent school children, involvement in bully-victim problems, and perceived social support. Suicide Life Threat Behav. 1999;29(2):119-130. 10407965

[11] Sullivan MK Homophobia, history, and homosexuality: trends for sexual minorities. J Hum Behav Soc Environ. 2004;8(2-3):1-13. doi:10.1300/J137v08n02_01

[12] Kann L, Olsen EOM, McManus T, Sexual identity, sex of sexual contacts, and health-related behaviors among students in grades 9-12: United States and selected sites, 2015. MMWR Surveill Summ. 2016:65(SS-9):1-202. doi:10.15585/mmwr.ss6509a1 27513843

[13] American Psychological Association Guidelines for psychological practice with transgender and gender nonconforming people. Am Psychol. 2015;70(9):832-864. doi:10.1037/a0039906 26653312

[14] Goldblum P, Testa RJ, Pflum S, Hendricks ML, Bradford J, Bongar B The relationship between gender-based victimization and suicide attempts in transgender people. Prof Psychol Res Pr. 2012;43(5):468-475. doi:10.1037/a0029605

[15] Earnshaw VA, Bogart LM, Poteat VP, Reisner SL, Schuster MA Bullying among lesbian, gay, bisexual, and transgender youth. Pediatr Clin North Am. 2016;63(6):999-1010. doi:10.1016/j.pcl.2016.07.004 27865341 PMC8941671

[16] Pi J Transgender in China. J LGBT Youth. 2010;7(4):346-358. doi:10.1080/19361653.2010.512518

[17] Parkin S LGBT rights-focused legal advocacy in China: the promise, and limits, of litigation. Fordham Int Law J. 2018;41:1243.

[18] Hu X, Wang Y LGB identity among young Chinese: the influence of traditional culture. J Homosex. 2013;60(5):667-684. doi:10.1080/00918369.2013.773815 23593953

[19] Yang X, Zhao L, Wang L, Quality of life of transgender women from China and associated factors: a cross-sectional study. J Sex Med. 2016;13(6):977-987. doi:10.1016/j.jsxm.2016.03.369 27117528

[20] Wei C-z, Liu W-l The association between school bullying and mental health of sexual minority students. Chin J Clin Psychol. 2015;23(4):701-705.

[21] Cheng FK Dilemmas of Chinese lesbian youths in contemporary mainland China. Sex Cult. 2018;22(1):190-208. doi:10.1007/s12119-017-9460-8

[22] Shao J, Chang ES, Chen C The relative importance of parent-child dynamics and minority stress on the psychological adjustment of LGBs in China. J Couns Psychol. 2018;65(5):598-604. doi:10.1037/cou0000281 29999331

[23] Nock MK, Green JG, Hwang I, Prevalence, correlates, and treatment of lifetime suicidal behavior among adolescents: results from the National Comorbidity Survey Replication Adolescent Supplement. JAMA Psychiatry. 2013;70(3):300-310. doi:10.1001/2013.jamapsychiatry.55 23303463 PMC3886236

[24] Haas AP, Rodgers PL, Herman JL Suicide attempts among transgender and gender non-conforming adults. https://williamsinstitute.law.ucla.edu/research/suicide-attempts-among-transgender-and-gender-non-conforming-adults/. Accessed July 30, 2019.

[25] Meyer IH Minority stress and mental health in gay men. J Health Soc Behav. 1995;36(1):38-56. doi:10.2307/2137286 7738327

[26] Meyer IH Prejudice, social stress, and mental health in lesbian, gay, and bisexual populations: conceptual issues and research evidence. Psychol Bull. 2003;129(5):674-697. doi:10.1037/0033-2909.129.5.674 12956539 PMC2072932

[27] Allport GW The Nature of Prejudice. Reading, MA: Addison-Wesley; 1954.

[28] Lehavot K, Simoni JM The impact of minority stress on mental health and substance use among sexual minority women. J Consult Clin Psychol. 2011;79(2):159-170. doi:10.1037/a0022839 21341888 PMC4059829

[29] Hendricks ML, Testa RJ A conceptual framework for clinical work with transgender and gender nonconforming clients: an adaptation of the Minority Stress Model. Prof Psychol Res Pr. 2012;43(5):460-467. doi:10.1037/a0029597

[30] Zhu X, Gao Y, Gillespie A, Health care and mental wellbeing in the transgender and gender-diverse Chinese population. Lancet Diabetes Endocrinol. 2019;7(5):339-341. doi:10.1016/S2213-8587(19)30079-8 30902476

[31] Chen R, Zhu X, Wright L, Suicidal ideation and attempted suicide amongst Chinese transgender persons: national population study. J Affect Disord. February 2019;245:1126-1134. doi:10.1016/j.jad.2018.12.011 30699856

[32] Jin H, Chen Z, Guo F, Zhang J, Yang Y, Wang Q A short Chinese version of Center For Epidemiologic Studies Depression Scale. Chin J of Behav Med and Brain Sci. 2013;22(12):1133-1136.

[33] Wang Y-Y, Dong M, Zhang Q, Suicidality and clinical correlates in Chinese men who have sex with men (MSM) with HIV infection. Psychol Health Med. 2019;24(2):137-143. doi:10.1080/13548506.2018.1515495 30175922

[34] Simons L, Schrager SM, Clark LF, Belzer M, Olson J Parental support and mental health among transgender adolescents. J Adolesc Health. 2013;53(6):791-793. doi:10.1016/j.jadohealth.2013.07.019 24012067 PMC3838484

[35] Pedro KT, Esqueda MC Exploring school victimization and weapon carrying among military-connected lesbian, gay, bisexual, and transgender youth in California schools [published online July 25, 2017]. J Interpers Violence. doi:10.1177/0886260517719537 29294847

[36] Greytak EA, Kosciw JG, Diaz EM Harsh Realities: The Experiences of Transgender Youth in Our Nation’s Schools. New York, NY: Gay, Lesbian and Straight Education Network; 2009.

[37] Toomey RB, Ryan C, Diaz RM, Card NA, Russell ST Gender-nonconforming lesbian, gay, bisexual, and transgender youth: school victimization and young adult psychosocial adjustment. Dev Psychol. 2010;46(6):1580-1589. doi:10.1037/a0020705 20822214

[38] Rostosky SS, Owens GP, Zimmerman RS, Riggle ED Associations among sexual attraction status, school belonging, and alcohol and marijuana use in rural high school students. J Adolesc. 2003;26(6):741-751. doi:10.1016/j.adolescence.2003.09.002 14643744

[39] Collier KL, van Beusekom G, Bos HM, Sandfort TG Sexual orientation and gender identity/expression related peer victimization in adolescence: a systematic review of associated psychosocial and health outcomes. J Sex Res. 2013;50(3-4):299-317. doi:10.1080/00224499.2012.750639 23480074 PMC3602930

[40] Murdock TB, Bolch MB Risk and protective factors for poor school adjustment in lesbian, gay, and bisexual (LGB) high school youth: variable and person-centered analyses. Psychol Sch. 2005;42(2):159-172. doi:10.1002/pits.20054

[41] Goodenow C Classroom belonging among early adolescent students: relationships to motivation and achievement. J Early Adolesc. 1993;13(1):21-43. doi:10.1177/0272431693013001002

[42] Eisenberg ME, Neumark-Sztainer D, Perry CL Peer harassment, school connectedness, and academic achievement. J Sch Health. 2003;73(8):311-316. doi:10.1111/j.1746-1561.2003.tb06588.x 14593947

[43] Hatchel T, Valido A, De Pedro KT, Huang Y, Espelage DL Minority stress among transgender adolescents: the role of peer victimization, school belonging, and ethnicity [published online June 25, 2018]. J Child Fam Stud. doi:10.1007/s10826-018-1168-3

[44] Reisner SL, Biello KB, White Hughto JM, Psychiatric diagnoses and comorbidities in a diverse, multicity cohort of young transgender women: baseline findings from project LifeSkills. JAMA Pediatr. 2016;170(5):481-486. doi:10.1001/jamapediatrics.2016.0067 26999485 PMC4882090

[45] Toomey RB, Syvertsen AK, Shramko M Transgender adolescent suicide behavior. Pediatrics. 2018;142(4):e20174218. doi:10.1542/peds.2017-4218 30206149 PMC6317573

[46] Becerra-Culqui TA, Liu Y, Nash R, Mental health of transgender and gender nonconforming youth compared with their peers. Pediatrics. 2018;141(5):e20173845. doi:10.1542/peds.2017-3845 29661941 PMC5914494

[47] Tebbe EA, Moradi B Suicide risk in trans populations: an application of minority stress theory. J Couns Psychol. 2016;63(5):520-533. doi:10.1037/cou0000152 27089059

[48] Liu XC, Ma DD, Kurita H, Tang MQ Self-reported depressive symptoms among Chinese adolescents. Soc Psychiatry Psychiatr Epidemiol. 1999;34(1):44-47. doi:10.1007/s001270050110 10073120

[49] Reisner SL, Vetters R, Leclerc M, Mental health of transgender youth in care at an adolescent urban community health center: a matched retrospective cohort study. J Adolesc Health. 2015;56(3):274-279. doi:10.1016/j.jadohealth.2014.10.264 25577670 PMC4339405

[50] Perez-Brumer A, Day JK, Russell ST, Hatzenbuehler ML Prevalence and correlates of suicidal ideation among transgender youth in California: findings from a representative, population-based sample of high school students. J Am Acad Child Adolesc Psychiatry. 2017;56(9):739-746. doi:10.1016/j.jaac.2017.06.010 28838578 PMC5695881

