# Supplementary material for: Self-reported Rates of Abuse, Neglect, and Bullying Experienced by Transgender and Gender-Nonbinary Adolescents in China
Source: JAMA Netw Open. 2019 Sep 6;2(9):e1911058. doi: 10.1001/jamanetworkopen.2019.11058 (PMC6735403; doi:10.1001/jamanetworkopen.2019.11058)
Supplement: Supplement. — eTable. Survey Questions on Abuse, Neglect, and Bullying at Home and in School, Translated From Chinese [file jamanetwopen-2-e1911058-s001.pdf]

# Supplementary Online Content

Peng K, Zhu X, Gillespie A, et al. Self-reported rates of abuse, neglect, and bullying experienced by transgender and gender-nonbinary adolescents in China. *JAMA Netw Open*. 2019;2(9):e1911058. doi:10.1001/jamanetworkopen.2019.11058

**eTable.** Survey Questions on Abuse, Neglect, and Bullying at Home and in School, Translated From Chinese

This supplementary material has been provided by the authors to give readers additional information about their work.

© 2019 Peng K et al. *JAMA Network Open.*

eTable. Survey Questions on Abuse, Neglect, and Bullying at Home and in School, Translated From Chinese

| Have you experienced any of the following from your parents or guardians?[1] |
|---|
| • Economic control |
| • Being forced to change my gender expression |
| • Deliberately neglecting/evading my gender identity and giving me no care or support |
| • Verbal abuse/insults |
| • Restricting personal freedom |
| • Physical assault |
| • Persistent neglect |
| • Eviction from the home/Cutting off contact |
| • Being coerced or forced to undergo conversion therapy |
| • Being forced to have sex with others |

| Have you experienced any of the following from your peers or teachers? |
|---|
| • Verbal bullying |
| • Being publicly mocked |
| • Isolation/exclusion |
| • Spread of rumors |
| • Threats or intimidation |
| • Cyberbullying via social media |
| • Physical abuse |

[1] Note: Participants were asked to additionally report number of occurrences in past 12 months

© 2019 Peng K et al. *JAMA Network Open.*
